# Transcriptome analysis reveals rapid defence responses in wheat induced by phytotoxic aphid *Schizaphis graminum* feeding

**DOI:** 10.1186/s12864-020-6743-5

**Published:** 2020-05-04

**Authors:** Yong Zhang, Yu Fu, Qian Wang, Xiaobei Liu, Qian Li, Julian Chen

**Affiliations:** State Key Laboratory for Biology of Plant Diseases and Insect Pests, Institute of Plant Protection, Chinese Academy of Agricultural Sciences, Beijing, 100193 People’s Republic of China

**Keywords:** *Schizaphis graminum*, Transcriptomics analysis, Chlorophyll content, Defence responses, Hydrogen peroxide accumulation, NADPH oxidase

## Abstract

**Background:**

*Schizaphis graminum* is one of the most important and devastating cereal aphids worldwide, and its feeding can cause chlorosis and necrosis in wheat. However, little information is available on the wheat defence responses triggered by *S. graminum* feeding at the molecular level.

**Results:**

Here, we collected and analysed transcriptome sequencing data from leaf tissues of wheat infested with *S. graminum* at 2, 6, 12, 24 and 48 hpi (hours post infestation). A total of 44,835 genes were either up- or downregulated and differed significantly in response to aphid feeding. The expression levels of a number of genes (9761 genes) were significantly altered within 2 hpi and continued to change during the entire 48 h experiment. Gene Ontology analysis showed that the downregulated DEGs were mainly enriched in photosynthesis and light harvesting, and the total chlorophyll content in wheat leaves was also significantly reduced after *S. graminum* infestation at 24 and 48 hpi. However, a number of related genes of the salicylic acid (SA)-mediated defence signalling pathway and MAPK-WRKY pathway were significantly upregulated at early feeding time points (2 and 6 hpi). In addition, the gene expression and activity of antioxidant enzymes, such as peroxidase and superoxide dismutase, were rapidly increased at 2, 6 and 12 hpi. DAB staining results showed that *S. graminum* feeding induced hydrogen peroxide (H_2_O_2_) accumulation at the feeding sites at 2 hpi, and increased H_2_O_2_ production was detected with the increases in aphid feeding time. Pretreatment with diphenylene iodonium, an NADPH oxidase inhibitor, repressed the H_2_O_2_ accumulation and expression levels of SA-associated defence genes in wheat.

**Conclusions:**

Our transcriptomic analysis revealed that defence-related pathways and oxidative stress in wheat were rapidly induced within hours after the initiation of aphid feeding. Additionally, NADPH oxidase plays an important role in aphid-induced defence responses and H_2_O_2_ accumulation in wheat. These results provide valuable insight into the dynamic transcriptomic responses of wheat leaves to phytotoxic aphid feeding and the molecular mechanisms of aphid-plant interactions.

## Background

Plants have been interacting with herbivores for millions of years and have evolved a variety of defence mechanisms against herbivory, such as constitutive defences and inducible defences [1, 2]. Constitutive defences are physical barriers such as cell walls, waxy cuticles, and bark, protecting the plant from attacks [3–6]. Inducible defences include the rapid detection of herbivory by plants through specific recognition and signalling systems and the production of a range of products or secondary metabolites that are toxic, repellent or anti-digestive to herbivores [7–9]. Some herbivory-induced products are volatile organic compounds (VOCs) released by plants that can attract the natural enemies of herbivores, resulting in an indirect means of protection [2, 10–12].

Several phytohormones, including jasmonic acid (JA), salicylic acid (SA), ethylene (ET), abscisic acid (ABA), auxin, and cytokinins, are key mediators of plant defences [13–18]. JA and SA and their derivatives play a predominant role in modulating plant defences against pests and pathogens, respectively [2, 19].

The JA-dependent signalling pathway is usually activated in response to leaf-chewing herbivores, cell-content feeders and necrotrophic pathogens [20–22]. The SA-mediated defence pathway is primarily induced by piercing-sucking herbivores and biotrophic pathogens [21]. Hemipterans have highly modified piecing-sucking mouthparts (stylets) that follow an intercellular pathway and feed on phloem sap from sieve elements (SEs) [23]. Feeding of hemipterans, like that of whiteflies and aphids, causes minimum mechanical damage in plant cells during feeding and mainly induces SA-dependent signalling defence pathway by suppressing the JA-associated defence pathway [24].

The greenbug, *Schizaphis graminum*, is one of the most important and devastating cereal aphids in the world, damaging plants by feeding on phloem sap and serving as a vector for transmitting viruses, such as barley yellow dwarf virus (BYDV) [25, 26]. With global warming, the potential risk of *S. graminum* infestations will increase, especially in the northern hemisphere, which could increase global food insecurity and poverty by destroying economically important crops [27]. In contrast to most other aphid species, *S. graminum* is a phytotoxic aphid, and its feeding can rapidly induce leaf chlorosis in susceptible plants, resulting in the deterioration of plant quality and even plant death. Previous studies have demonstrated that *S. graminum* feeding induced SA- and JA-dependent defence pathways in sorghum (*Sorghum bicolor*) [28], and reactive oxygen species (ROS) levels, peroxidase (POD) and laccase activities were also increased in switchgrass (*Panicum virgatum*) after *S. graminum* feeding [29]. However, few studies have been conducted to identify the defence mechanisms in wheat in response to *S. graminum* feeding, and the mechanisms underlying the induction of damage by *S. graminum* infestation are still unclear. In the present study, we investigated the dynamic wheat responses to *S. graminum* feeding by integrating results from high-throughput RNA sequencing and cytological examination to uncover the mechanism underlying the induction of defence responses and damage symptoms by phytotoxic aphid feeding.

## Results

### Transcriptomic analysis of wheat leaves in response to *S. graminum* at different time points

Global transcriptomic changes in response to phytotoxic aphid feeding were examined in leaves of wheat seedlings infested with *S. graminum* at 2, 6, 12, 24 and 48 hpi. A total of 166.41 Gb of clean data were obtained from the 18 leaf samples, and each of these samples contained ≥7.6 Gb of data with Q30 quality scores ≥92.32% (Additional file 1). Subsequently, for each sample between 44.3 and 59.5 million reads were mapped uniquely, with roughly 5% of the total reads mapping to multiple locations (Additional file 2).

**Fig. 1:**
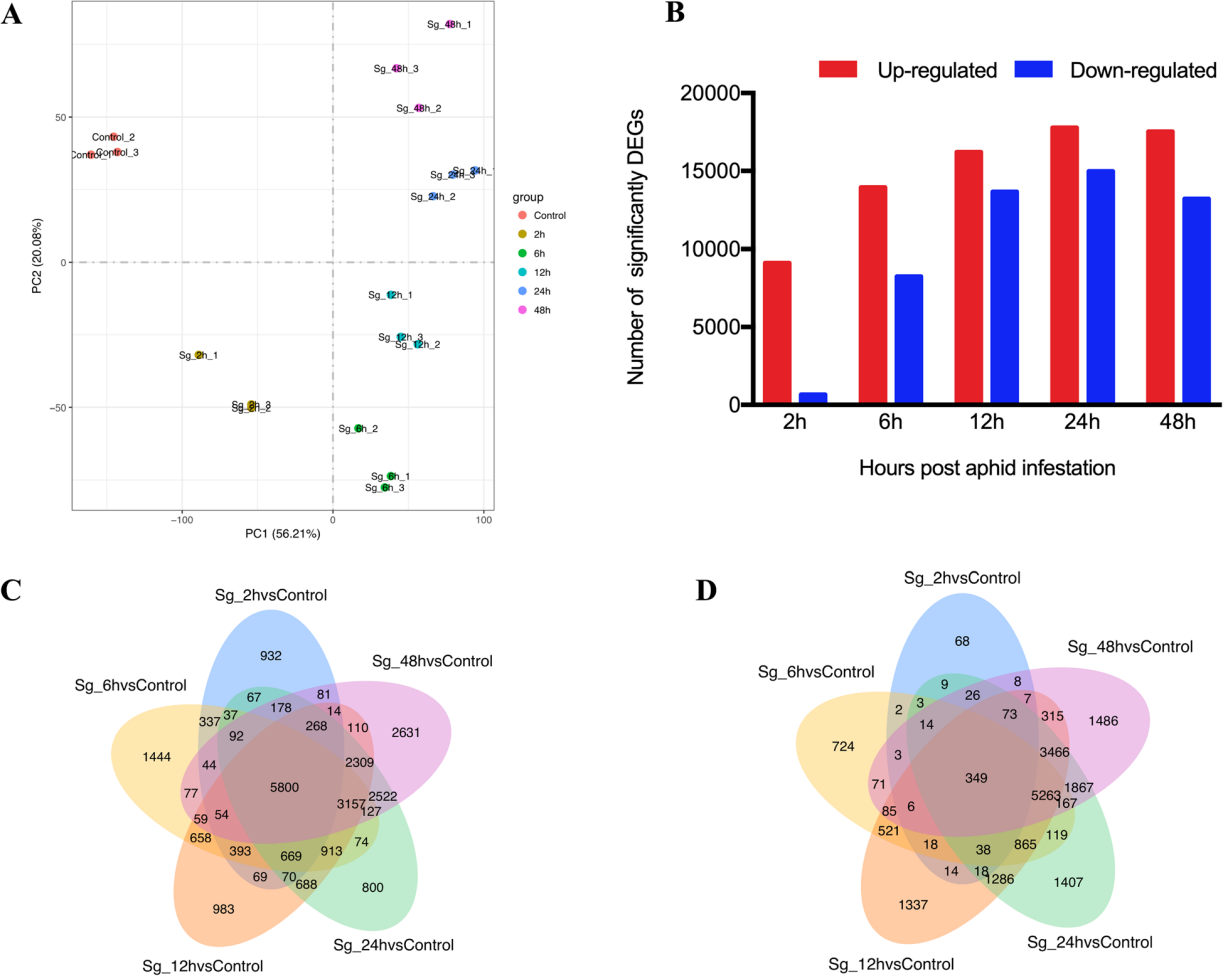
Transcriptomic overview of a time course of *S. graminum* feeding on wheat leaves. **a**: PCA plot of global transcriptome profiles. **b**: Total number of transcripts that were significantly up- or downregulated in response to aphid feeding. **c**, **d**: Venn diagram illustrating the number of genes up- or downregulated by aphid feeding over the time course. *P* < 0.01 FDR and Log_2_ FC ≥ 1 or ≤ − 1

The gene expression levels were used to conduct a PCA for each of the biological replicates. Each replicate from the same group was clustered closely together, which suggested that the repeatability of each treatment was satisfactory, and the samples from different time points of *S. graminum* infestation were clustered far from each other and the control groups, which indicated that aphid feeding induced significant changes in gene expression (Fig. 1a).

The *P* value≤0.01 (false discovery rate [FDR] adjusted) and Log_2_-fold change (Log_2_FC) ≥1 or ≤ − 1 were set as thresholds for DEGs in wheat leaves at different time points. Then, these identified DEGs were used for further analysis. A total of 44,835 DEGs were identified in wheat leaves at different time points (2, 6, 12, 24 and 48 hpi) of aphid feeding (Additional files 3, 4, 5). Briefly, 9761 (9105 up- and 656 downregulated), 22,183 (13,935 up- and 8248 downregulated), 29,875 (16,214 up- and 13,661 downregulated), 32,741 (17,771 up- and 14,970 downregulated) and 30,729 (17,523 up- and 13,206 downregulated) DEGs were identified at 2, 6, 12, 24 and 48 hpi, respectively (Fig. 1b, Additional file 4). The distribution of up- and downregulated genes was calculated for each time point and are presented in a Venn diagram (Fig. 1c and d). Although a unique set of genes increased at each time point (total 74,548), the expression levels of a large number of genes (5800) were significantly upregulated at all time points. In addition, a unique set of genes was significantly downregulated at each time point (total 50,741), and only 349 genes showed decreased expression at all five time points.

### Gene ontology (GO) analysis of DEGs

**Fig. 2:**
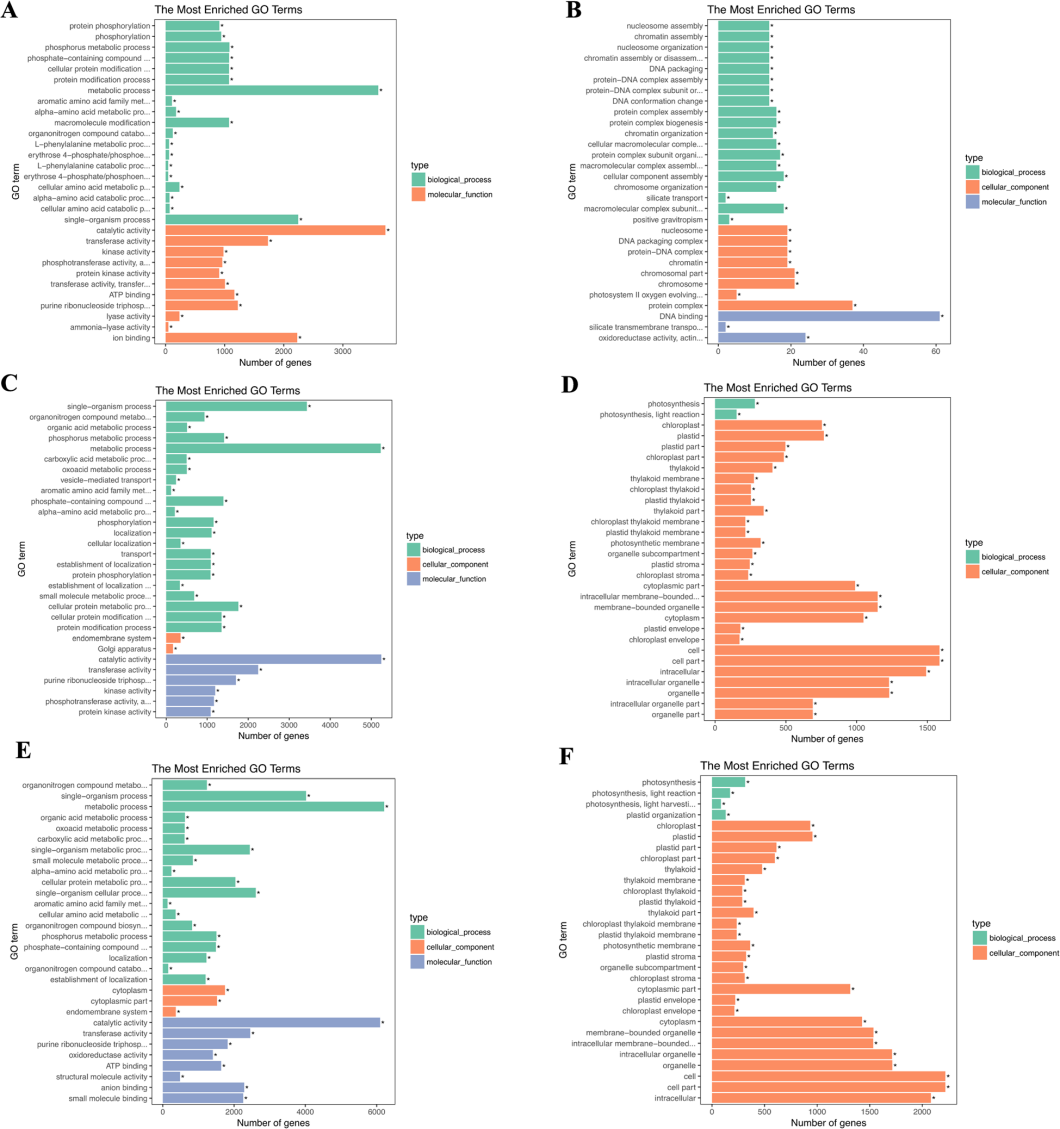
GO enrichment analysis of the differentially expressed genes (DEGs) in wheat leaves in response to *S. graminum* feeding at 2, 6 and 12 hpi. **a**: GO enrichment analysis of upregulated DEGs at 2 hpi; **b**: GO enrichment analysis of downregulated DEGs at 2 hpi; **c**: GO enrichment analysis of upregulated DEGs at 6 hpi; **d**: GO enrichment analysis of downregulated DEGs at 6 hpi; **e**: GO enrichment analysis of upregulated DEGs at 12 hpi; **f**: GO enrichment analysis of downregulated DEGs at 12 hpi

GO analysis was used for the functional classification of the DEGs in wheat leaves after aphid infestation. The top 30 enriched GO terms of all DEGs are shown in Additional file 6. GO analysis of DEGs induced by *S. graminum* feeding at early time points is shown in Fig. 2. At 2 hpi (Fig. 2a, b), within the biological process category, the upregulated DEGs were mainly enriched in metabolic process, single-organism process and phosphorus metabolic process. Within the molecular function category, the largest proportion of upregulated DEGs induced by the aphid feeding was enriched in catalytic activity and transferase activity. At 6 and 12 hpi (Fig. 2c-f), the majority of the upregulated DEGs activated by the aphid feeding were enriched in metabolic processes and single-organism processes within the biological process category, protein kinase activity and phosphotransferase activity. In the molecular function category, the upregulated DEGs were mainly enriched in catalytic activity and transferase activity.

At 6 and 12 hpi (Fig. 2c-f), within the biological process category, the downregulated DEGs were mainly enriched in photosynthesis and light reaction. In the cellular component group, greater percentages of DEGs in the cellular component category were enriched in cell and cell parts. In addition, many downregulated DEGs at 6 and 12 hpi were also enriched in chloroplasts, further indicating the damage in wheat leaves caused by *S. graminum* feeding.

### Chlorophyll content in wheat leaves after *S. graminum* feeding

Transcriptome analysis showed that aphid feeding negatively affected the photosynthetic processes of wheat, and the transcript levels of many light-harvesting- and photosystem-associated genes, such as *ribulose-1,5-bisphosphate carboxylase*, *chlorophyll a-b binding proteins*, *ferredoxin thioredoxin reductase*, and *PsbP family proteins*, were significantly downregulated (Table 1).

**Table 1:** DEGs associated with plant photosynthesis process in wheat leaves in response to *S. graminum* feeding

Gene Description	Gene ID	Log_2_ Fold Change				
		2 h	6 h	12 h	24 h	48 h
*Ribulose-1,5-bisphosphate carboxylase*	TraesCS2B01G079100	/	−2.49	−3.46	−5.92	− 5.84
	TraesCS2D01G065100	/	−2.33	−3.28	−5.81	−5.76
	TraesCS2B01G079500	/	−2.93	−3.71	−6.02	−7.35
	TraesCS2A01G066900	/	−2.30	−3.58	−5.54	−5.40
	TraesCS2B01G079400	/	−2.21	−2.72	−4.28	−3.53
	TraesCS2A01G067300	/	−2.52	−3.24	−5.38	−5.02
	TraesCS2A01G067200	/	−2.57	−3.66	−5.72	−6.11
	Novel03072	/	−3.14	−3.67	−6.83	−7.70
*Chlorophyll A-B binding protein*	TraesCS1B01G432700	−1.31	−2.79	−3.09	−6.07	−7.87
	TraesCS1A01G403300	−1.34	−3.05	−3.47	−5.46	−6.92
	TraesCS2A01G204800	/	−3.73	−3.87	−4.99	−5.43
	TraesCS7D01G276300	/	−3.40	−3.10	−6.97	−8.54
	TraesCS2B01G233400	/	−3.47	−4.31	−6.50	−7.98
	TraesCS5D01G464800	/	−6.20	−5.85	−8.98	−11.86
*Ferredoxin thioredoxin reductase*	TraesCS6A01G234900	/	−2.25	−2.44	−2.72	−1.61
	TraesCS6D01G217500	/	−2.10	−2.35	−3.20	−1.76
	TraesCS6B01G263600	/	−2.11	−2.54	−3.11	−1.88
*PsbP domain proteins*	TraesCS2D01G255100	/	−2.20	−2.94	−4.06	−3.35
	TraesCS2B01G267500	/	−2.27	−2.81	−3.66	−3.04
	TraesCS4B01G203100	/	−2.35	−3.78	−6.54	−5.95
	TraesCS4D01G204000	/	−2.21	−3.56	−5.61	−4.94
	TraesCS4A01G101500	/	−2.02	−3.46	−6.13	−4.44

“/” indicates no significant differences between aphid-infested and control groups

**Fig. 3:**
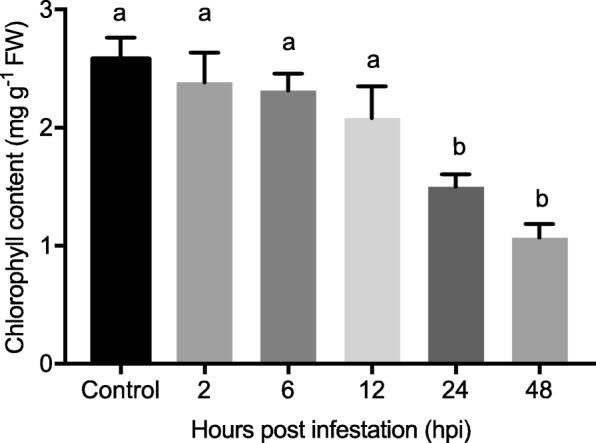
Chlorophyll content in wheat leaves after *S. graminum* feeding at 2, 6, 12, 24 and 48 hpi. The values are presented as the means ± SE of three biological replicates. Different letters indicate significant differences among treatments (*P* < 0.05, ANOVA)

The results in Fig. 3 suggested that the total chlorophyll content in wheat leaves at 2, 6 and 12 hpi was not significantly different from that of the control. However, the total chlorophyll content was significantly decreased to 1.49 ± 0.10 mg g^− 1^ FW after 24 h of aphid feeding (F_5,18_ = 9.447, *P* = 0.0001) and was further reduced to 1.07 ± 0.11 mg g^− 1^ FW at 48 hpi, which was significantly lower than that of the control (2.58 ± 0.18 mg g^− 1^ FW).

### Transcript levels of genes involved in SA- and JA-dependent defence pathways in wheat leaves after *S. graminum* feeding

Phytohormone metabolic pathways are commonly used by plants for defence against both pests and pathogens. The transcriptome data in Table 2 showed that all six *phenylalanine ammonia-lyase* (*PAL*) genes involved in SA biosynthesis were significantly upregulated in response to *S. graminum* at different time points, and the expression levels of *PAL* gradually decreased with increased aphid feeding time (4.96 to 16.16-fold). Furthermore, *PR* genes that respond to SA were also significantly upregulated during all time points of aphid feeding (4.92 to 20.59-fold).

**Table 2:** DEGs involved in jasmonic acid and salicylic acid synthesis pathways in response to *S. graminum* feeding at different time points

Plant hormone	Gene Description	Gene ID	Log_2_ Fold Change				
			2 h	6 h	12 h	24 h	48 h
Salicylic acid	*PAL*	TraesCS2A01G196700	9.89	9.20	7.91	6.65	4.95
		TraesCS2B01G224300	11.68	10.40	8.77	8.26	7.07
		TraesCS6A01G222700	11.71	11.34	10.28	8.26	6.65
		TraesCS1B01G122800	11.53	10.96	9.77	8.82	7.25
		TraesCS2D01G204400	12.13	11.84	10.52	10.09	8.95
		TraesCS2B01G224000	16.16	15.31	14.42	14.10	12.39
	*PR Proteins*	TraesCS7D01G161200	12.23	17.10	19.59	20.59	19.85
		TraesCS5A01G183300	17.13	12.41	15.17	15.99	15.03
		TraesCS5B01G181500	6.40	11.09	14.29	15.29	14.80
		Novel10567	4.92	6.09	6.80	6.59	6.50
		TraesCS5B01G442900	8.34	10.02	10.96	11.18	10.23
Jasmonic acid	*LOX*	TraesCS4B01G037700	5.78	3.56	3.46	3.74	3.59
		TraesCS7D01G244800	7.76	9.25	8.26	6.93	5.259
		TraesCS2B01G333600	3.98	3.35	2.46	2.07	1.49
		TraesCS7B01G145200	5.89	/	/	/	/
	*FAD*	TraesCS4A01G109300	6.77	6.46	5.22	5.07	4.50
		TraesCS2D01G279500	1.62	3.29	2.80	2.23	1.36
		TraesCS6B01G309400	1.48	1.38	/	/	/
		TraesCS6A01G280000	1.87	1.37	/	/	−1.20
		TraesCS5A01G123600	/	/	−2.04	−2.45	−2.45
	*AOC*	TraesCS6D01G314300	2.84	3.16	2.61	2.50	1.53
		TraesCS6B01G365200	2.27	2.31	1.43	1.04	/
		TraesCS6A01G334800	2.52	2.60	2.16	2.37	2.03
	*JAR1*	TraesCS3A01G145300	/	−2.31	−2.46	−3.41	− 2.74

“/” indicates no significant differences between aphid-infested and control groups

A greater effect on genes involved in JA metabolism was observed over time (Table 2). Three *lipoxygenase* (*LOX*) genes were significantly upregulated by *S. graminum* feeding at different time points (1.49 to 9.25-fold), and one *lipoxygenase* (*LOX*) was only upregulated at 2 hpi (5.89-fold). The expression levels of *allene oxide cyclase* (*AOC*) were also significantly increased at various aphid feeding time points. In contrast, the *jasmonic acid-amido synthetases* (*JARs*) were downregulated in infested plants (− 2.31 to − 3.41-fold). There were also five *fatty acid desaturase* (*FAD*) genes that had variable expression levels, with the majority being upregulated (1.36 to 6.77-fold) during infestation. However, some were also downregulated during the three later time points (− 1.20 to − 2.45-fold), which suggests that the expression of *FAD* genes may be fine-tuned during defence responses.

The mitogen-activated protein kinase (MAPK) cascade is a key signalling pathway of plant defence, and WRKY transcription factors (TFs) appear to be regulated by MAPKs and involved in the regulation of plant defence. The transcript levels of several MAPKs were significantly upregulated (1.01- to 3.48-fold) in response to *S. graminum* feeding at 12, 24 and 48 hpi. Additionally, several WRKY TFs in wheat leaves were significantly induced (1.59 to 11.14-fold) in response to aphid feeding at different time points (Table 3).

**Table 3:** DEGs involved in MAPK-WRKY pathways in response to *S. graminum* feeding at different time points

Gene Description	Gene ID	Log_2_ Fold Change				
		2 h	6 h	12 h	24 h	48 h
*MAPKs*	Novel11623	1.24	1.44	/	1.32	1.29
	TraesCS4D01G198600	/	/	3.48	3.39	3.20
	TraesCS3D01G225600	/	/	2.30	2.26	1.25
	TraesCS4B01G197800	/	/	2.53	2.66	2.67
	TraesCS7B01G322900	/	/	2.58	2.64	2.48
	TraesCS4A01G106400	/	/	2.35	2.74	2.8
	TraesCS7B01G309900	/	/	1.64	1.64	1.52
	TraesCS7A01G422500	/	/	1.57	1.18	1.01
*WRKY*	Novel00700	4.12	5.28	5.36	7.17	6.40
	Novel01914	3.56	4.82	6.04	7.42	6.56
	Novel05138	6.51	8.40	9.81	11.06	11.14
	Novel01125	/	4.86	5.24	6.50	5.74
	Novel08030	/	2.66	2.30	2.29	1.59

“/” indicates no significant differences between aphid-infested and control groups

### Effects of *S. graminum* feeding on hydrogen peroxide (H_2_O_2_) accumulation and the activity of antioxidant enzymes in wheat leaves

As shown in Table 4, *S. graminum* feeding upregulated various ROS-scavenging genes, such as *POD* and *SOD* (*superoxide dismutase*), at 2 hpi. The expression levels of these two genes were increased at 6, 12 and 24 hpi and then gradually decreased at 48 hpi, but the transcript levels were still significantly increased compared with the control levels. The expression levels of *catalase* (*CAT*) genes showed no significant differences at 2 hpi compared to the control levels. Among them, two genes were significantly downregulated at 6, 12, 24 and 48 hpi, and the other two genes were significantly upregulated.

**Table 4:** DEGs associated with ROS scavenging in wheat leaves in response to *S. graminum* feeding at different time points

Gene Description	Gene ID	Log_2_ Fold Change				
		2 h	6 h	12 h	24 h	48 h
*Plant peroxidase*	TraesCS2B01G125200	12.80	15.52	15.72	14.65	12.48
	TraesCS2A01G107500	13.98	16.53	16.85	15.85	13.60
	TraesCS2A01G107700	14.56	15.56	15.14	13.36	11.37
	TraesCS2B01G124800	12.93	16.87	17.34	16.67	14.75
	TraesCS2D01G107800	12.95	15.37	15.70	14.63	12.43
*Superoxide dismutase*	TraesCS2D01G123300	2.07	3.81	3.55	2.83	2.72
	TraesCS2A01G121200	1.96	3.85	3.24	2.53	2.70
	Novel03118	1.56	3.02	2.65	2.09	2.35
*Catalase*	TraesCS5A01G498000	/	−1.52	−1.97	−2.57	−1.77
	TraesCS6B01G330700	/	−1.80	−1.53	−2.19	−1.86
	TraesCS6A01G041700	/	3.38	5.90	4.63	4.65
	TraesCS6D01G048300	/	1.64	3.83	3.37	3.39

“/” indicates no significant differences between aphid-infested and control groups

**Fig. 4:**
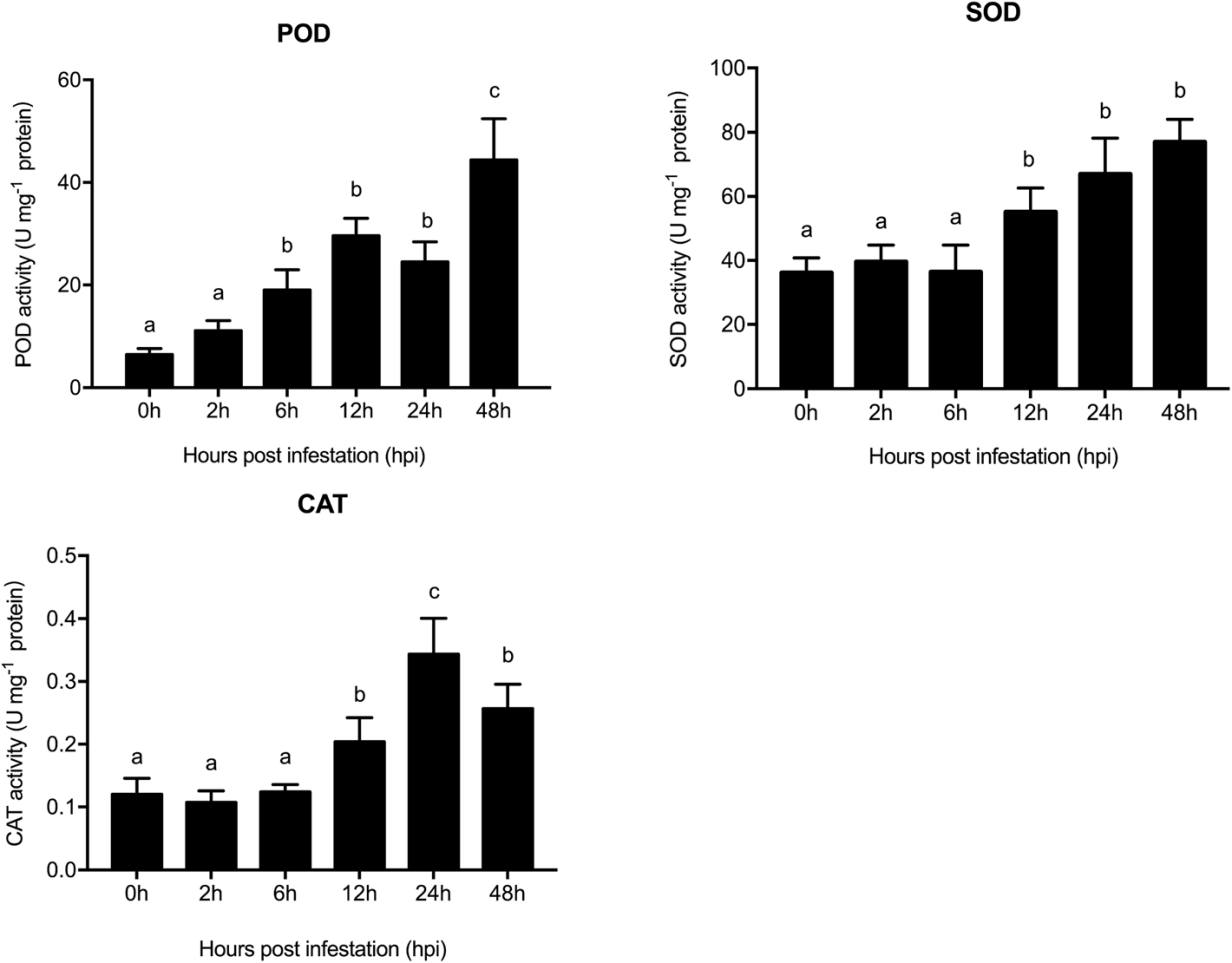
Activity of the antioxidant enzymes POD, SOD and CAT in wheat leaves in response to *S. graminum* feeding. The values are presented as the means ± SE of three biological replicates. Different letters indicate significant differences among treatments (*P* < 0.05, ANOVA)

The activities of three antioxidant enzymes, POD, SOD and CAT, were also examined in wheat leaves infested with aphids (Fig. 4). Compared with the control, the activity of POD was significantly increased after 6 h (19.01 ± 3.94 U mg^− 1^ protein) of *S. graminum* feeding and reached a peak at 48 hpi (44.44 ± 3.37 U mg^− 1^ protein; F_5, 12_ = 10.17, *P* = 0.001). Furthermore, the activities of SOD (55.10 ± 7.55 U mg^− 1^ protein; F_5,12_ = 5.15, *P* = 0.009) and CAT (0.20 ± 0.038 U mg^− 1^ protein; F_5,12_ = 7.27, *P* = 0.002;) were significantly upregulated after 12 h of *S. graminum* feeding. The increased activity of ROS scavengers induced by aphid feeding suggested that *S. graminum* feeding induces oxidative stress in wheat leaves.

**Fig. 5:**
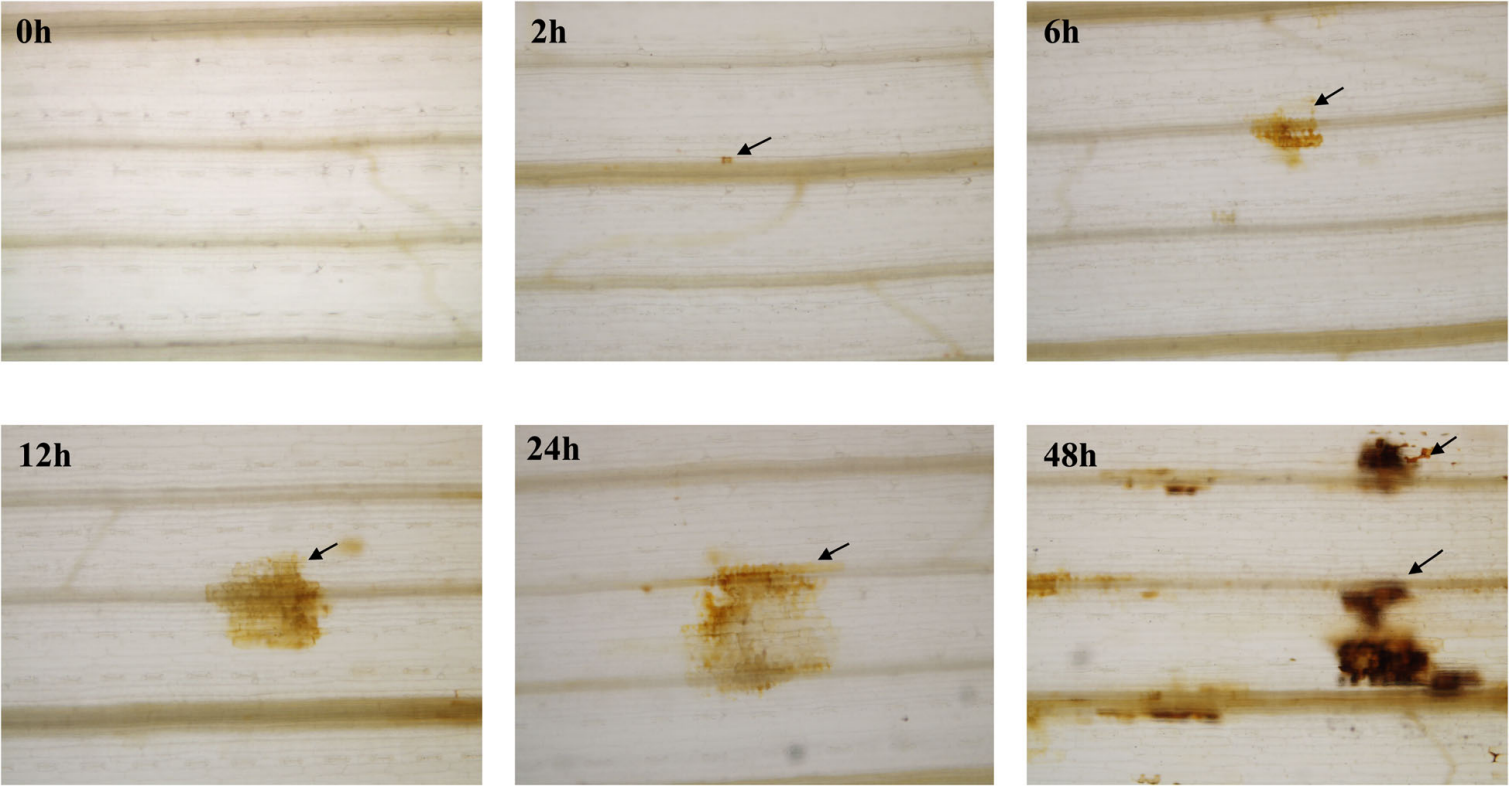
Detection of H_2_O_2_ accumulation in wheat leaves in response to *S. graminum* feeding at different time points using DAB staining. Images are representative of three biological replicates

To further examine the effects of aphid feeding on oxidative stress in wheat, *S. graminum*-infested leaves were examined after cytological staining with 3,3′-diaminobenzidine (DAB), which was used to detect the production of H_2_O_2_. As shown in Fig. 5, small and obvious brown spots were detected at 2 hpi, indicating H_2_O_2_ accumulation at the aphid feeding site. The number and size of the spots increased with increasing time aphid feeding time.

### Effects of inhibition of NADPH oxidases on H_2_O_2_ accumulation and defence responses in wheat leaves

**Fig. 6:**
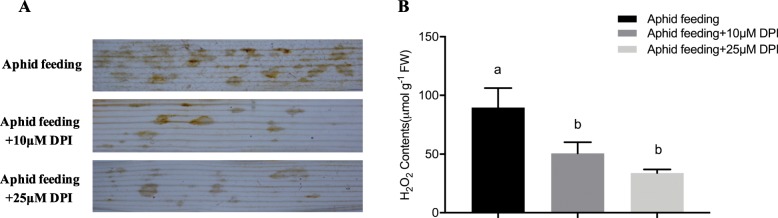
Effects of pretreatment with diphenylene iodonium (DPI), an NADPH oxidase inhibitor, on the accumulation (**a**) and contents of H_2_O_2_ (**b**) induced by *S. graminum* feeding in wheat leaves. Images are representative of three biological replicates

To detect the roles of plasma membrane NADPH oxidases in H_2_O_2_ accumulation induced by *S. graminum* feeding, wheat leaves were treated with the NADPH oxidase inhibitor diphenylene iodonium (DPI). The DAB staining results, shown in Fig. 6a, indicate that many obvious brown spots were detected at the aphid feeding sites of infested leaves, but fewer brown spots were observed after 10 μM and 25 μM DPI treatments, indicating that the H_2_O_2_ production induced by aphid feeding was inhibited by DPI. Additionally, the H_2_O_2_ contents were significantly decreased to 50.60 ± 9.51 μmol g^− 1^ FW and 33.93 ± 3.00 μmol g^− 1^ FW in wheat leaves treated with 10 μM and 25 μM DPI, respectively (F_2,6_ = 6.44, *P* = 0.032) (Fig. 6b).

**Fig. 7:**
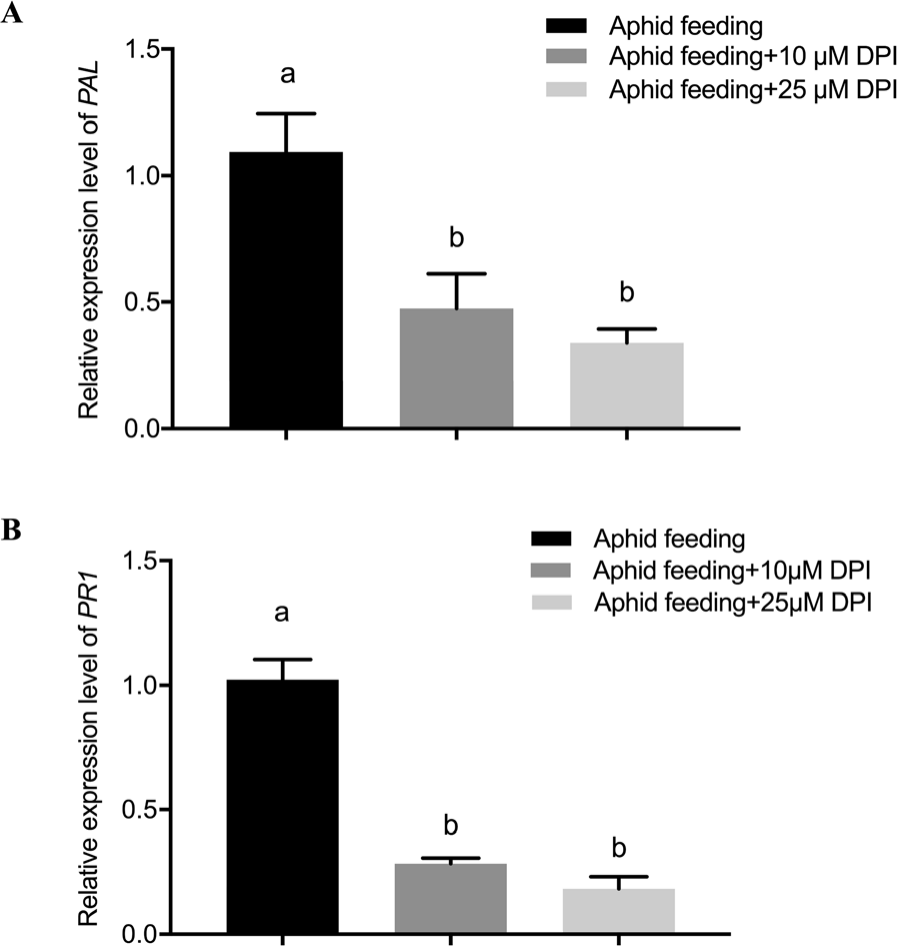
Effects of pretreatment with DPI on the expression levels of the SA-associated defence genes *PAL* (**a**) and *PR1* (**b**) induced by *S. graminum* feeding in wheat leaves. The values are presented as the means ± SE of three biological replicates. Different letters indicate significant differences among treatments (*P* < 0.05, ANOVA)

As shown in Fig. 7a and b, DPI treatment had significant effects on the expression levels of defence response genes in wheat leaves. The expression levels of the salicylic acid-related genes *PAL* and *PR1* were significantly reduced in DPI-treated wheat leaves compared to the control (wheat leaves infested with aphids for 24 h) (F_2,6_ = 10.96, *P* = 0.01; F_2,6_ = 65.53, *P* < 0.001).

## Discussion

### Wheat transcriptomes are rapidly and significantly regulated in response to *S. graminum* feeding

Plants usually activate rapid responses to biotic and abiotic stress. For example, in *Arabidopsis*, rapid and highly localized increases in calcium was detected around the feeding sites of *M. persicae* after just a few minutes of feeding [30]. More than 500 genes associated with the primary metabolism and defence responses were significantly upregulated in peach leaves after 3 h of infestation by *M. persicae* [31]. Almost 800 genes were upregulated after 3 h of aphid feeding in maize [32]. Similarly, rapid and strong physiological responses were activated in wheat by *S. graminum* feeding in our study. The transcript levels of more than 9000 genes involved in various physiological processes were significantly up- or downregulated after just 2 h of aphid feeding. A previous study demonstrated that severe degenerative changes were detected in vascular cells adjacent to the stylet path of susceptible wheat plants as early as 1 h after *S. graminum* infestation using transmission electron microscopy [33]. Cellular damage caused by aphid feeding may be responsible for the rapid and strong physiological changes in wheat leaves.

### *S. graminum* feeding results in damage to chlorophyll and suppression of photosynthesis

As a typical phytotoxic aphid, *S. graminum* feeding induced obvious chlorosis in the aphid-susceptible wheat variety [34]. Consistent with previous studies [35], the total contents of chlorophyll were significantly reduced in wheat leaves after aphid infestation. Additionally, transcriptomic analysis showed that many genes involved in photosynthesis, such as *ribulose-1,5-bisphosphate carboxylase* and *chlorophyll A-B binding protein,* were significantly downregulated after *S. graminum* feeding, and downregulated DEGs at early time points of aphid feeding (after 6 h) were enriched in photosynthesis and light harvesting pathways, indicating that *S. graminum* feeding imposed rapid damage to chlorophyll and then led to a reduction in the photosynthetic activity of the plants.

### Defence signalling pathway induced in wheat following challenge with *S. graminum*

Piercing-sucking hemipteran insects, such as aphids and whiteflies, mainly induce SA-mediated defence signal pathways [36]. However, some studies have also demonstrated that genes involved in both the JA and SA defence response pathways, such as *LOX*, *PIs*, *PAL*, and *PR1*, are significantly upregulated by aphid feeding [37–40]. Similarly, we found that *S. graminum* feeding significantly increased the expression levels of several genes related to the SA and JA signalling pathways. The fold changes in the expression levels of *PAL* and *PR1* were very high at various aphid feeding times, suggesting that a strong SA defence pathway was activated by *S. graminum* feeding, which might be responsible for the induction of chlorosis in wheat. However, the genes involved in the JA signalling pathway had variable expression levels, with the majority being upregulated during infestation but some also being downregulated during the later time points, which suggests that the expression of *FAD* genes may be fine-tuned during defence responses. Several *JAR1* (jasmonic acid resistant 1) genes were also significantly downregulated by *S. graminum*. JAR1 is a JA-aminosynthetase that is required to activate JA in *Arabidopsis* [41]. These results suggested that although JA-responsive genes were upregulated, increased JA levels or the JA defence pathway may not be activated by *S. graminum* feeding. Zhang et al. demonstrated that the SA contents in wheat leaves after *S. graminum* feeding were significantly greater than those found in wheat leaves without aphid infestation, but no significant changes were detected for the JA contents in aphid-infested wheat leaves [35].

### Protein phosphatases and MAPK-WRKY pathways are activated in response to *S. graminum* feeding

At different time points of aphid feeding, many upregulated genes were enriched in protein phosphorylation. Protein phosphatases are one of the most predominant post-translational modifications (PTMs) and play a central role in signal transduction through the phosphorylation and de-phosphorylation of proteins in eukaryotes [42]. A large body of evidence demonstrates that phosphorylation is essential for immune responses in plants [43]. For example, in *Arabidopsis*, more than 1170 phosphopeptides from 472 phosphoproteins were identified after treatments with flg22 or xylanase, both of which elicit immune responses in *Arabidopsis* cell cultures [44]. A total of 109 differentially phosphorylated residues of membrane-associated proteins on activation of the intracellular RPS2 receptor were identified by phosphoproteomic screening using an inducible expression system of the bacterial effector avrRpt2 in *Arabidopsis* [45]. Additionally, phosphorylation plays a central role in the progression of the signal through the MAP kinase cascade, which includes a class of protein kinases that plays a crucial signalling role in plant defence against pathogen and herbivore attacks [46]. The expression levels of several MAPKs and mitogen-activated protein kinase kinases (MAPKKs) were significantly upregulated by *S. graminum* feeding in wheat, indicating that MAPK signalling plays critical roles in regulating the induced defence responses of *S. graminum*.

The expression levels of many WRKY genes were significantly upregulated by *S. graminum* feeding. WRKY transcription factors comprise a large protein family sharing a DNA binding domain of approximately 60 amino acids that contains an invariable sequence WRKYGQK and a zinc-finger-like domain [47, 48]. An increasing number of WRKY TFs have been identified as substrates of MAPKs and important components in MAPK signalling pathways for the regulation of plant immunity [49–51]. For instance, in response to *Botrytis cinerea* infection, WRKY33 is phosphorylated and activated by MPK3/MPK6, which induce downstream defence responses in *Arabidopsis*, such as ethylene production [52]. Phospho-mimicking mutants of WRKY transcription factors are involved in the induction of RBOHB-dependent ROS burst and cell death in *Nicotiana benthamiana*, suggesting that WRKYs are associated with the induction of HR-cell death as MAPK substrates in plants [53]. Whether the MAPK-WRKY pathway is involved in the cell death and chlorosis caused by *S. graminum* is still unknown.

### *S. graminum* feeding induces strong oxidative stress in wheat

Abiotic and biotic stresses generally induce the accumulation of ROS, such as H_2_O_2_ and O^2^-, and cause oxidative stress in plants. H_2_O_2_ is one of the most important ROS in plant-pathogen and plant-herbivore interactions and has an important role in signal transmission and plant defence responses [54, 55]. The production of ROS, such as hydrogen peroxide (H_2_O_2_), rapidly occurs in fern plants after just 1 h of herbivory [56, 57]. In infested barley plants, the maximum level of H_2_O_2_ was found just approximately 20 min after aphid infestation [58]. In our study, rapid H_2_O_2_ accumulation was also detected in wheat after *S. graminum* feeding, which suggested that aphid infestation results in strong oxidative stress. In plant cells, antioxidant enzymes, such as CAT, POD, and SOD keep ROS at low concentrations, avoiding oxidative damage while allowing them to play crucial functions in signal transduction [59]. Excess cellular levels of ROS cause damage to proteins, nucleic acids, lipids, membranes and organelles, which can lead to activation of cell death processes such as apoptosis. The transcriptomic and enzymatic results in our study showed that *S. graminum* feeding increased the gene expression levels and enzyme activities of ROS scavengers in wheat leaves. It is speculated that although ROS scavengers were significantly upregulated by *S. graminum*, H_2_O_2_ production might exceed the cellular antioxidant capacity, resulting in oxidative damage to cellular components and chlorosis in leaves.

Strong SA-dependent defence responses were triggered by *S. graminum*. Considerable evidence demonstrates that H_2_O_2_ can interact with plant defence hormones, such as SA [60]. For example, SA treatment can enhance H_2_O_2_ levels, and H_2_O_2_ has been proposed to function downstream of SA in plants based on the evidence that SA can participate in regulating antioxidant enzymes, such as CAT, SOD and ascorbate peroxidase (APx) [61–63]. In contrast, other studies report that elevated H_2_O_2_ levels could activate SA biosynthesis via stimulation of BA2H (benzoic acid 2-hydroxylase) activity in tobacco cells [64]. Furthermore, other studies have indicated that H_2_O_2_ does not function downstream of SA in regulating PR protein expression [65]. The mechanisms by which SA interacts with H_2_O_2_ production in plants remain unclear.

### NADPH oxidases are involved in the regulation of *S. graminum*-induced H_2_O_2_ accumulation and defence responses of wheat

Plasma-membrane-localized NADPH oxidases, known as respiratory burst oxidase homologues (Rbohs), are membrane-bound enzyme complexes that are important components for H_2_O_2_ generation and plant immunity [66]. A lack of RBOH expression leads to very low levels of ROS production, resulting in the alteration of different plant responses in terms of cell death and pathogen resistance [67–69]. To further examine the roles of NADPH oxidases in H_2_O_2_ accumulation and defence responses induced by *S. graminum* feeding, wheat leaves were treated with the NADPH oxidase inhibitor DPI at different concentrations. H_2_O_2_ production and the expression levels of SA-dependent defence genes were inhibited, suggesting the involvement of NADPH oxidases in H_2_O_2_ accumulation and defence responses induced by *S. graminum* feeding. Phosphorylation at specific conserved residues in RBOHD is also required for ROS production in both PAMP-triggered immunity (PTI) and effector-triggered immunity (ETI) and immunity against avirulent bacteria and a virulent necrotrophic fungus [70]. Whether RBOHD phosphorylation is associated with the ROS production, defence responses and chlorosis induced by *S. graminum* feeding in wheat is worthy of further study.

## Conclusions

In conclusion, the transcriptomic profiling of wheat performed in this study revealed dynamic physiological changes in wheat leaves in response to phytotoxic aphid *S. graminum* feeding. *S. graminum* feeding triggered rapid plant defence responses and ROS-scavenging activities in wheat plants. A cytological analysis showed that substantial H_2_O_2_ accumulated in wheat leaves in response to *S. graminum* feeding. Our results also demonstrated that NADPH oxidases play vital roles in the induction of H_2_O_2_ accumulation and SA-dependent defence responses triggered by *S. graminum* feeding. Our future studies will focus on the mechanisms of chlorosis induction by *S. graminum* feeding and the roles of salivary proteins of aphids in the induction of symptoms in plants.

## Methods

### Plants and aphids

Wheat seeds (*Triticum aestivum* var. Zhongmai175, Shengyuan Seed Industry Technology Co., Ltd., China) were immersed and germinated in sterilized Petri dishes with distilled water for 3–4 days at a temperature of 25 ± 1 °C and a photoperiod of 16:8 (L:D) h. After germination, healthy seedlings were selected and transferred into plastic plots containing organic soil and were grown in a climate chamber with a temperature of 20 ± 1 °C, a relative humidity of 40–60% and a photoperiod of L: D = 16 h: 8 h. All *S. graminum* were initiated from a single parthenogenetic female collected from a wheat field in Langfang city, Hebei Province, northern China. A clonal aphid lineage was maintained on wheat plants (Zhongmai175) under laboratory conditions at 20 ± 1 °C and 65 ± 10% relative humidity with a photoperiod of 14: 10 (L: D) h.

### Wheat plant infested with aphids

At the two-leaf stage (12-day-old), twenty 3rd instar *S. graminum* were placed into clip cages on the second leaves of wheat plants. Aphids were gently removed from wheat leaves using a soft brush at different time points after aphid feeding. Leaf tissues at the aphid feeding sites of each plant were collected into a 1.5 ml centrifuge tube using sterilized scissors, transferred to liquid nitrogen immediately and stored at − 80 °C until use. Two leaf tissues were collected from two independent plants with the same treatment to form one independent biological replicate. Three independent biological replicates were conducted for each treatment. Plants with empty clip cages were set as control groups.

### RNA extraction, library construction, and RNA sequencing

Total RNA from wheat leaves was extracted with TRIzol reagent (Invitrogen, Carlsbad, CA, USA) according to the manufacturer’s instructions. The RNA quality and quantity were examined using a DS-11 spectrophotometer (DeNovix, DE, USA), and the RNA integrity was confirmed using an RNA Nano 6000 Assay Kit with an Agilent 2100 Bioanalyser (Agilent Technologies, Santa Clara, CA, USA). Only RNA samples with an RNA Integrity Number (RIN) ≥ 7.0 were used in the subsequent analysis. Libraries were constructed using the TruSeq Stranded mRNA LT Sample Prep Kit (Illumina, San Diego, CA, USA) following the protocols provided by the manufacturer and were sequenced on the Illumina sequencing platform (Illumina HiSeq 4000), and 150-bp paired-end reads were generated. There were three independent biological replicates for each treatment.

### RNA-seq data analysis

To obtain high-quality reads, the reads containing adaptor sequences, more than 10% ambiguous bases (noted as N) and low-quality bases (Qphred ≤20 bases account for more than 50% of the entire read length of the reads) were filtered. The resulting clean reads were then aligned to the wheat reference genome (IWGSC RefSeq v1.0 genome) using TopHat v2.0.12 with the default values [71, 72]. Transcript assembly was performed using Cufflinks v2.1.1 with the default parameters [73]. All transcripts were compared with gene models in the reference genome to identify novel genes expressed from previously intergenic regions (class code “u”) using Cuffcompare [74]. The gene expression levels in all the samples were calculated using fragments per kilobase of exon model per million mapped reads (FPKM), and the genes with more than 1 FPKM in at least one sample of wheat leaves were used for further analysis [75]. An adjusted *P* value (FDR < 0.01) and fold change (FC) ratio (|Log_2_ FC| ≥ 1) were used to determine the differentially expressed genes (DEGs) between aphid-infested and control leaves using DESeq R package. Principal component analysis (PCA) was performed using the DESeq2 package for clustering the samples based on gene expression patterns [76]. The heatmap of DEGs was clustered using pheatmap (version 1.0.8, http://cran.r-project.org/web/packages/pheatmap) package in R. Gene Ontology (GO) (http://geneontology.org/) enrichment analyses of the DEGs were performed to understand the biological significance of the DEGs using the GOseq R package, and GO terms were considered significantly enriched with a corrected *p*-value < 0.05 [77].

### Changes of total chlorophyll levels in wheat leaves after aphid infestation

Total chlorophyll content in aphid-infested wheat leaves at different time points was examined according to the method of Aron [78] with slight modifications. A total of 100 mg of fresh leaf tissues was ground to a fine powder in liquid nitrogen using a mortar and pestle. Chlorophyll was extracted with 5 mL of 80% acetone (v/v) at 4 °C for overnight. The homogenate was centrifuged at 4000 g for 10 min at 4 °C, and the supernatant was used for chlorophyll assay. Chlorophyll content of leaves was detected spectrophotometrically, by reading the absorbance at 645 and 663 nm (DU800, Beckman, USA).

### Detection of H_2_O_2_ accumulation in wheat leaves induced by aphid feeding

To detect H_2_O_2_ accumulation in wheat leaves induced by *S. graminum* feeding, 3,3′-diaminobenzidine (DAB) (Sigma, Germany) staining was performed using the protocols reported by Wang et al. [79] with some modifications. In brief, leaf segments infested with *S. graminum* were cut off using sterilized scissors, and then immediately immersed in 1 mg mL^− 1^ DAB solution (10 mmol L^− 1^ Na_2_HPO_4_, pH 3.8) and incubated in a dark chamber overnight at room temperature. Leaves were decolorized in 95% ethanol solution and hyalinized in saturated chloral hydrate. The stained leaves were then visualized using an Olympus BX-63 microscope (Olympus Corporation, Japan). The endogenous H_2_O_2_ content in the wheat leaves was determined according to the methods described by Ferguson et al. [80].

### Determination of antioxidant enzymes in wheat leaves after aphid infestation

To examine the activities of peroxidase (POD), superoxide dismutase (SOD) and catalase (CAT) in wheat leaves at all time points after aphid infestation, a total of 200 m g of fresh leaf tissues was ground into a fine powder in liquid nitrogen using a mortar and pestle. The powder was then immediately homogenized in 1.5 mL of ice-cooled 50 mM potassium phosphate buffer (pH = 7.8) containing 0.1 mM EDTA, 1 mM phenylmethylsulfonyl fluoride (PMSF) and 1.0% (w/v) polyvinylpyrrolidone. The homogenate was centrifuged at 15,000 g for 30 min at 4 °C, and supernatant was immediately collected as crude enzyme extract for further assay. The activities of POD, SOD, CAT were determined by following the changes in absorbance at 470 nm, 560 nm and 240 nm respectively according to previously described [81].

### RT-qPCR

Total RNA was extracted from leaves using TRIzol Reagent (Invitrogen) according to the manufacturer’s recommended protocols. The concentration of RNA was measured by a DS-11 Spectrophotometer (DeNovix, DE, USA). One microgram of total RNA was reverse-transcribed into first-strand cDNA with oligo dT primers using the EasyScript All-in-One First-Strand cDNA Synthesis SuperMix for RT-qPCR (TransGen Biotech) following the manufacturer’s instructions, and cDNA templates were stored at − 20 °C until use. RT-qPCR was performed using the same protocols as previously described [35]. The RT-qPCR protocol consisted of an initial heat activation step of 95 °C for 10 min, followed by 40 cycles of 95 °C for 15 s and 60 °C for 40 s. Three biological replicates were performed for each treatment, and each biological replicate consisted of three technical replicates.

### Wheat seedlings treated with DPI solution

The leaves of wheat seedlings were treated with 10 μM and 25 μM diphenylene iodonium (DPI, a NADPH oxidase inhibitor) solution or deionized water (control) for 24 h and then infested with *S. graminum* for 48 h. Detection of H_2_O_2_ accumulation and expression levels of genes in wheat leaves were conducted as described previously.

### Statistical analyses

All the data were analysed using SPSS Statistics 20.0 software (SPSS Inc., Chicago, IL., USA). Normality of distribution and homogeneity of variances were tested by Shapiro-Wilk’s test and Levene’s test respectively, and the differences among groups were examined through one-way analysis of variance (Duncan). *P* values less than 0.05 were considered statistically significant.

## Supplementary information

**Additional file 1. **Summary for the transcriptome of wheat in response to *S. graminum* feeding at different time points using Illumina RNA-seq.

**Additional file 2.** Summary of clean reads mapped to the reference wheat genome.

**Additional file 3. **All of the differentially expressed genes (DEGs) of wheat leaves in response to *S. graminum* feeding at 2, 6, 12, 24 and 48 hpi.

**Additional file 4. **Volcano plots of DEGs in wheat leaves induced by *S. graminum* feeding for 2, 6, 12, 24 and 48 h compared with control. Red spots represent up-regulated DEGs, green spots represent down-regulated DEGs and blue spots represent genes with no significant expression.

**Additional file 5. **Heatmap with hierarchical clustering dendrograms of DEGs in wheat leaves in response to *S. graminum* feeding at 0 (control), 2, 6, 12, 24 and 48 hpi. Red indicates higher expression values across treatment, and blue represents lower expression values across treatment.

**Additional file 6. **GO enrichment analysis of all of the DEGs of wheat leaves in response to *S. graminum* feeding at 2 (A), 6 (B), 12 (C), 24 (D) and 48 hpi (E).

## Data Availability

The datasets used and analyzed during the current study are available from the corresponding author on reasonable request.

## Abbreviations

ABA: Abscisic acid
AOC: Allene oxide cyclase
BYDV: Barley yellow dwarf virus
CAT: Catalase
DAB: 3,3'-diaminobenzidine
DPI: Diphenylene iodonium
ET: Ethylene
FAD: Fatty acid desaturase
FPKM: Fragments per kilobase of exon model per million mapped reads
H_2_O_2_: Hydrogen peroxide
JA: Jasmonic acid
JAR: Jasmonic acid-amido synthetases
LOX: Lipoxygenase
MAPK: Mitogen-activated protein kinase
MAPKKs: Mitogen activated protein kinase kinases
PCA: Principal component analysis
POD: Peroxidase
PAL: Phenylalanine ammonia-lyase
PTMs: Post-translational modifications
RNA-Seq: High-throughput RNA sequencing
ROS: Reactive oxygen species
SA: Salicylic acid
SEs: Sieve elements
VOCs: Volatile organic compounds
