# Supplementary material for: Transcriptome analysis reveals rapid defence responses in wheat induced by phytotoxic aphid Schizaphis graminum feeding
Source: BMC Genomics. 2020 May 4;21:339. doi: 10.1186/s12864-020-6743-5 (PMC7199342; doi:10.1186/s12864-020-6743-5)
Supplement: Supplementary file 1 — Additional file 1. Summary for the transcriptome of wheat in response to S. graminum feeding at different time points using Illumina RNA-seq. [file 12864_2020_6743_MOESM1_ESM.doc]

Table S1. Summary for the transcriptome of wheat in response to *S. graminum* feeding at different time points using Illumina RNA-seq.

| Sample name | Raw reads | Clean reads | Clean bases | Q20(%) | Q30(%) | GC content(%) |
| --- | --- | --- | --- | --- | --- | --- |
| 0h-1 | 67026614 | 65893356 | 9.88G | 97.19 | 92.77 | 55.73 |
| 0h-2 | 64649454 | 63422474 | 9.51G | 97.11 | 92.65 | 54.95 |
| 0h-2 | 62511782 | 61345006 | 9.2G | 97.07 | 92.54 | 55.27 |
| 2h-1 | 63002088 | 61132136 | 9.17G | 96.97 | 92.32 | 56.08 |
| 2h-2 | 61916100 | 60220612 | 9.03G | 97.07 | 92.55 | 55.25 |
| 2h-3 | 62740514 | 61101886 | 9.17G | 97.16 | 92.73 | 55.25 |
| 6h-1 | 51837066 | 50692990 | 7.6G | 97.14 | 92.66 | 55.76 |
| 6h-2 | 55578490 | 54289628 | 8.14G | 97.11 | 92.60 | 55.58 |
| 6h-3 | 60428900 | 58698560 | 8.8G | 97.11 | 92.62 | 55.70 |
| 12h-1 | 67585804 | 65781710 | 9.87G | 97.19 | 92.78 | 55.44 |
| 12h-2 | 67999794 | 64837000 | 9.73G | 97.16 | 92.75 | 55.81 |
| 12h-3 | 71089506 | 68812944 | 10.32G | 97.16 | 92.75 | 56.05 |
| 24h-1 | 65319770 | 63452118 | 9.52G | 97.36 | 92.71 | 55.85 |
| 24h-2 | 66414312 | 64537306 | 9.68G | 97.34 | 92.70 | 56.36 |
| 24h-3 | 61625674 | 60285084 | 9.04G | 97.50 | 93.03 | 55.78 |
| 48h-1 | 66282276 | 64040522 | 9.61G | 97.32 | 92.64 | 55.35 |
| 48h-2 | 55118882 | 53465048 | 8.02G | 97.25 | 92.47 | 55.34 |
| 48h-3 | 70066250 | 67474860 | 10.12G | 97.39 | 92.78 | 55.49 |
