# Supplementary material for: Transcriptome analysis reveals rapid defence responses in wheat induced by phytotoxic aphid Schizaphis graminum feeding
Source: BMC Genomics. 2020 May 4;21:339. doi: 10.1186/s12864-020-6743-5 (PMC7199342; doi:10.1186/s12864-020-6743-5)
Supplement: Supplementary file 2 — Additional file 2. Summary of clean reads mapped to the reference wheat genome. [file 12864_2020_6743_MOESM2_ESM.doc]

Table S2. Summary of clean reads mapped to the reference wheat genome

| Sample name | Total reads | Total mapped | Multiple mapped | Uniquely mapped |
| --- | --- | --- | --- | --- |
| 0h-1 | 65893356 | 60145347 (91.28%) | 3419935 (5.19%) | 56725412 (86.09%) |
| 0h-2 | 63422474 | 57575529 (90.78%) | 3231925 (5.1%) | 54343604 (85.69%) |
| 0h-2 | 61345006 | 55040763 (89.72%) | 3140736 (5.12%) | 51900027 (84.6%) |
| 2h-1 | 61132136 | 54765192 (89.58%) | 3202923 (5.24%) | 51562269 (84.35%) |
| 2h-2 | 60220612 | 54958762 (91.26%) | 3061120 (5.08%) | 51897642 (86.18%) |
| 2h-3 | 61101886 | 55757161 (91.25%) | 3085567 (5.05%) | 52671594 (86.2%) |
| 6h-1 | 50692990 | 46783871 (92.29%) | 2391986 (4.72%) | 44391885 (87.57%) |
| 6h-2 | 54289628 | 49656888 (91.47%) | 2657048 (4.89%) | 46999840 (86.57%) |
| 6h-3 | 58698560 | 53991083 (91.98%) | 2960965 (5.04%) | 51030118 (86.94%) |
| 12h-1 | 65781710 | 59806618 (90.92%) | 3185462 (4.84%) | 56621156 (86.07%) |
| 12h-2 | 64837000 | 59387850 (91.6%) | 3372361 (5.2%) | 56015489 (86.39%) |
| 12h-3 | 68812944 | 62909124 (91.42%) | 3399733 (4.94%) | 59509391 (86.48%) |
| 24h-1 | 63452118 | 58618689 (92.38%) | 3162389 (4.98%) | 55456300 (87.4%) |
| 24h-2 | 64537306 | 59490918 (92.18%) | 3195605 (4.95%) | 56295313 (87.23%) |
| 24h-3 | 60285084 | 55952351 (92.81%) | 2950027 (4.89%) | 53002324 (87.92%) |
| 48h-1 | 64040522 | 59336191 (92.65%) | 3350581 (5.23%) | 55985610 (87.42%) |
| 48h-2 | 53465048 | 49359314 (92.32%) | 2686372 (5.02%) | 46672942 (87.3%) |
| 48h-3 | 67474860 | 62163848 (92.13%) | 3586581 (5.32%) | 58577267 (86.81%) |
