# Supplementary figures and images for: Transcriptome analysis reveals rapid defence responses in wheat induced by phytotoxic aphid Schizaphis graminum feeding
Source: BMC Genomics. 2020 May 4;21:339. doi: 10.1186/s12864-020-6743-5 (PMC7199342; doi:10.1186/s12864-020-6743-5)

Sg\_2h vs Control

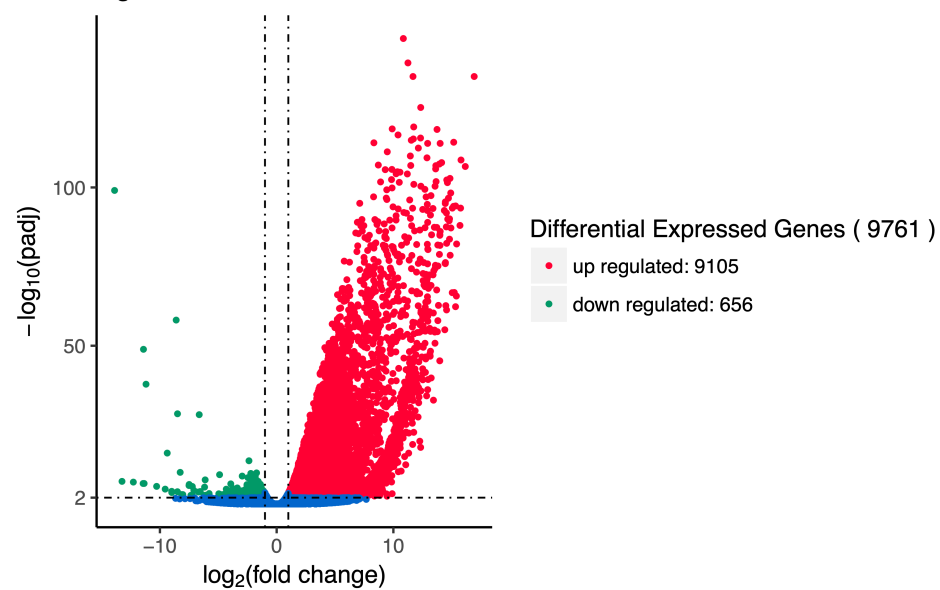

Sg\_6h vs Control

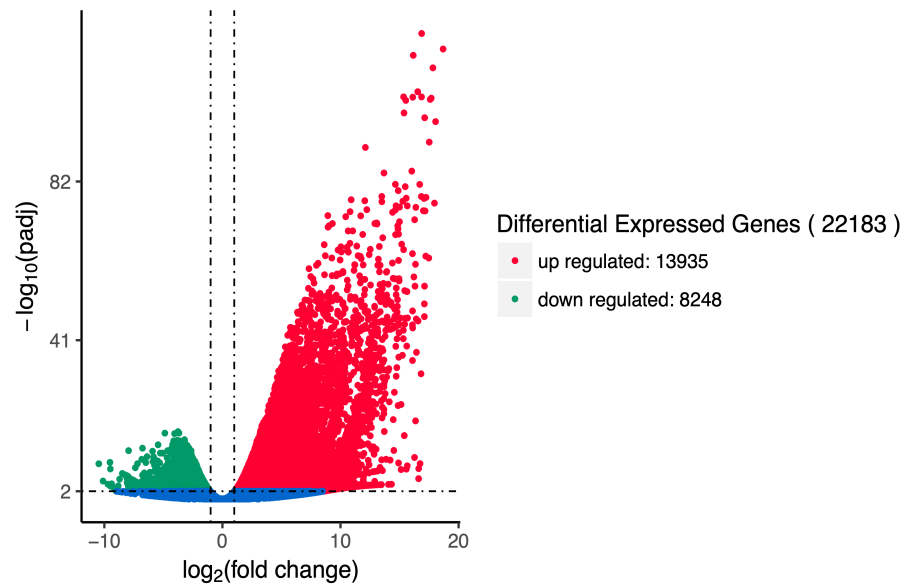

Sg\_12h vs Control

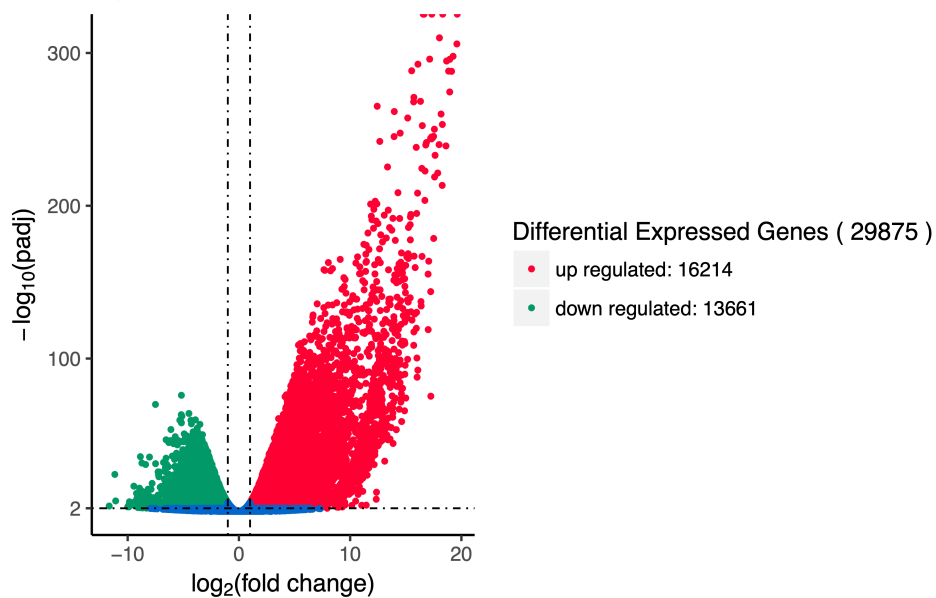

Sg\_24h vs Control

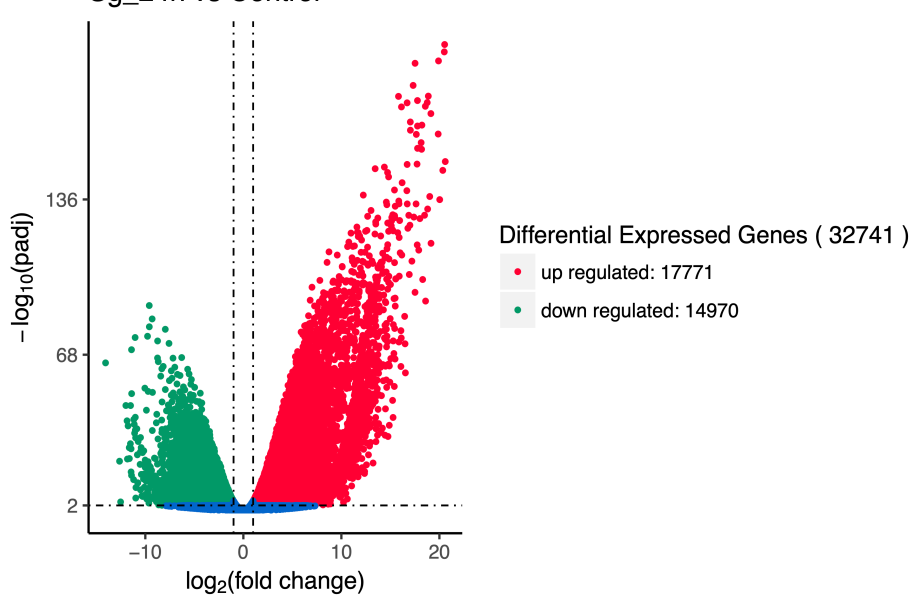

Sg\_48h vs Control

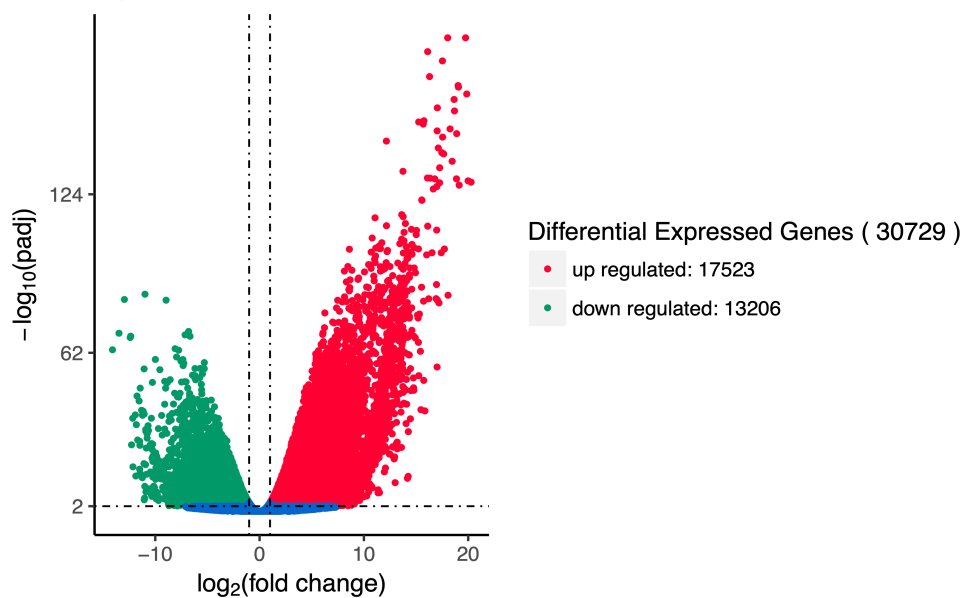

Supplement: Supplementary file 4 — Additional file 4. Volcano plots of DEGs in wheat leaves induced by S. graminum feeding for 2, 6, 12, 24 and 48 h compared with control. Red spots represent up-regulated DEGs, green spots represent down-regulated DEGs and blue spots represent genes with no significant expression. [file 12864_2020_6743_MOESM4_ESM.pdf]

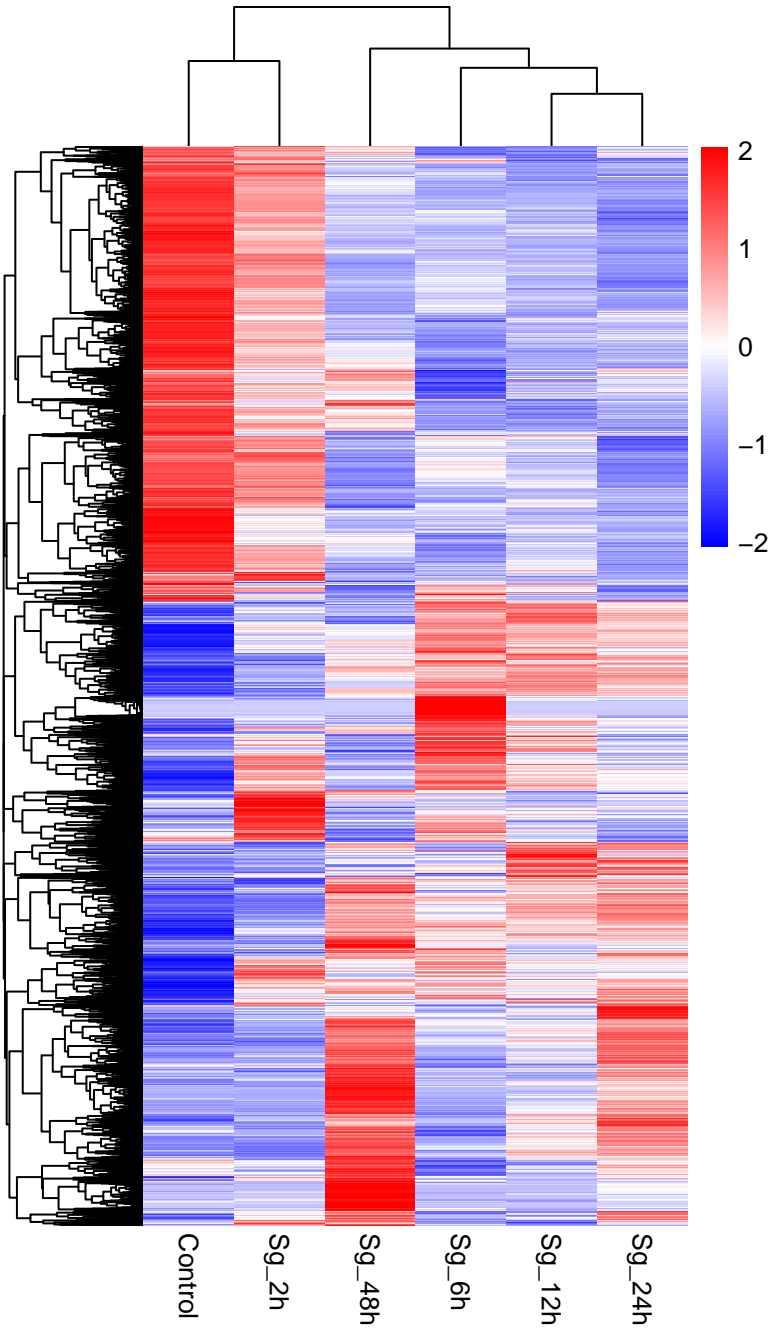

Supplement: Supplementary file 5 — Additional file 5. Heatmap with hierarchical clustering dendrograms of DEGs in wheat leaves in response to S. graminum feeding at 0 (control), 2, 6, 12, 24 and 48 hpi. Red indicates higher expression values across treatment, and blue represents lower expression values across treatment. [file 12864_2020_6743_MOESM5_ESM.pdf]

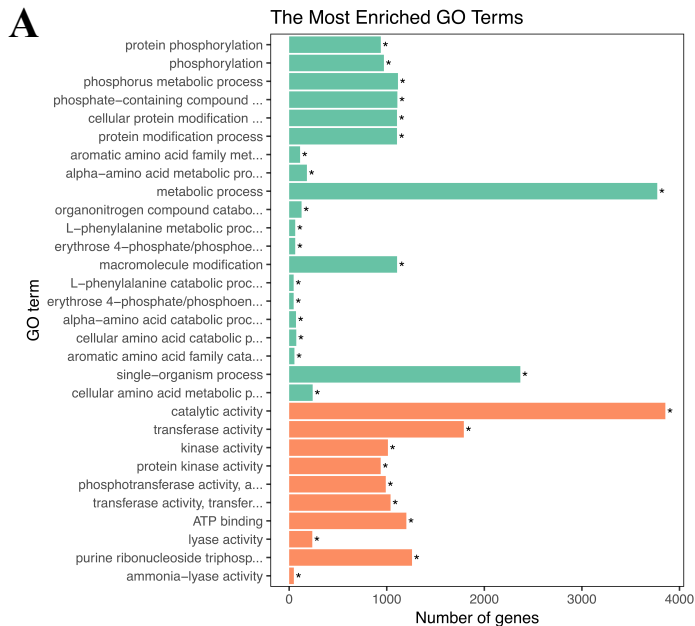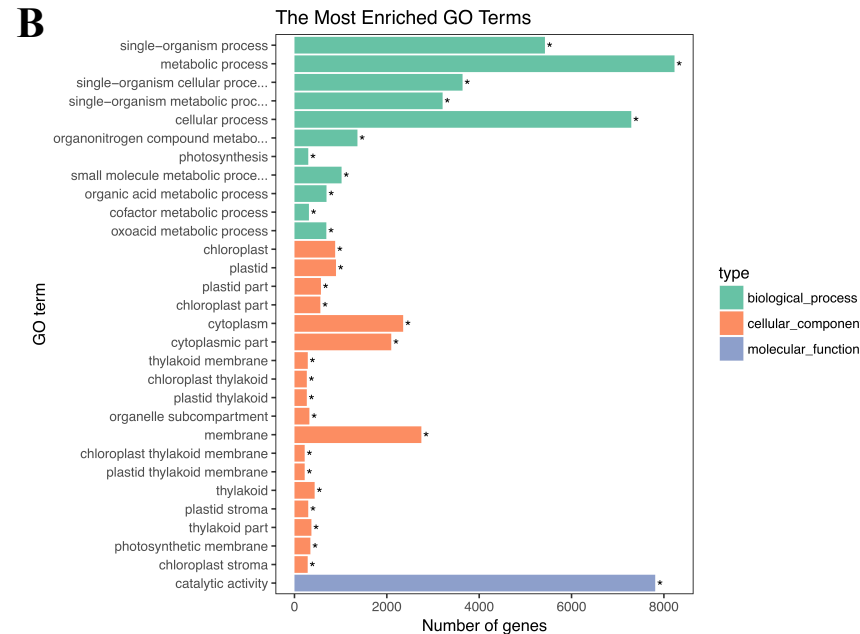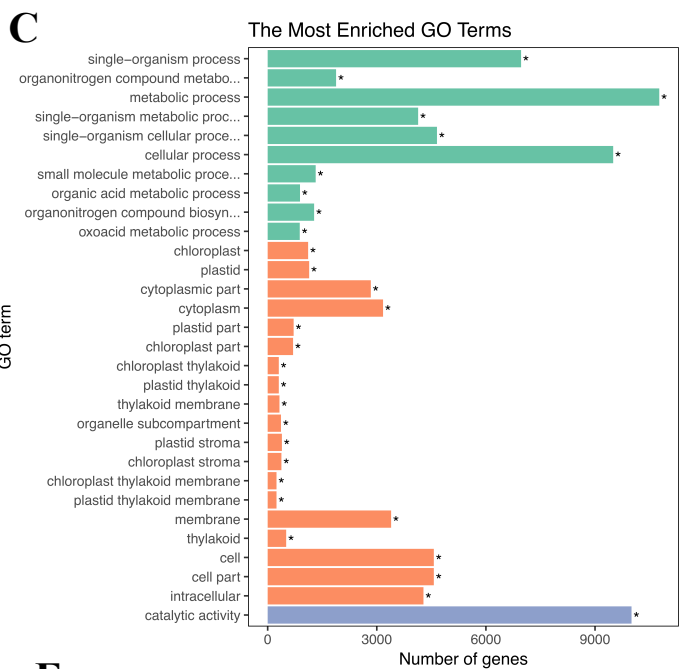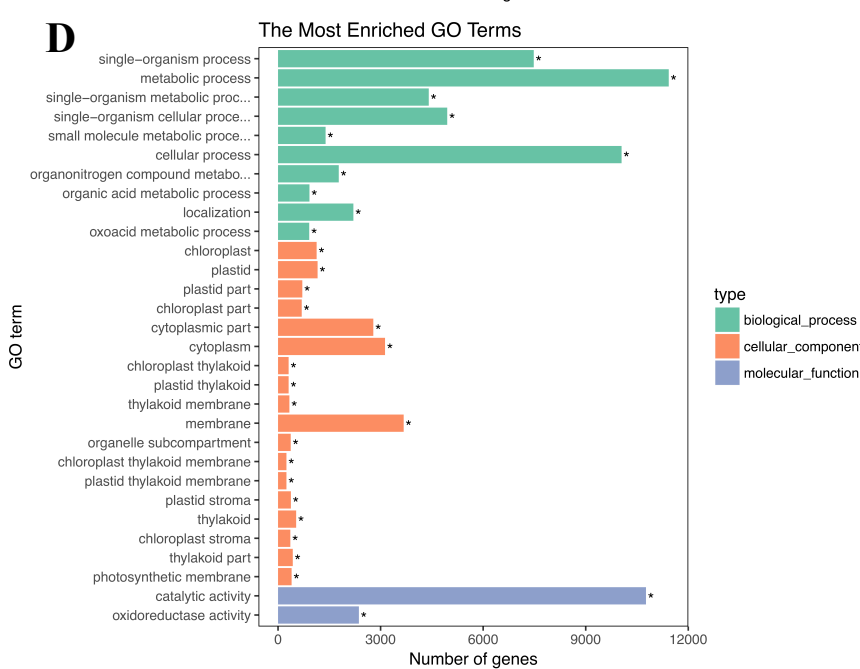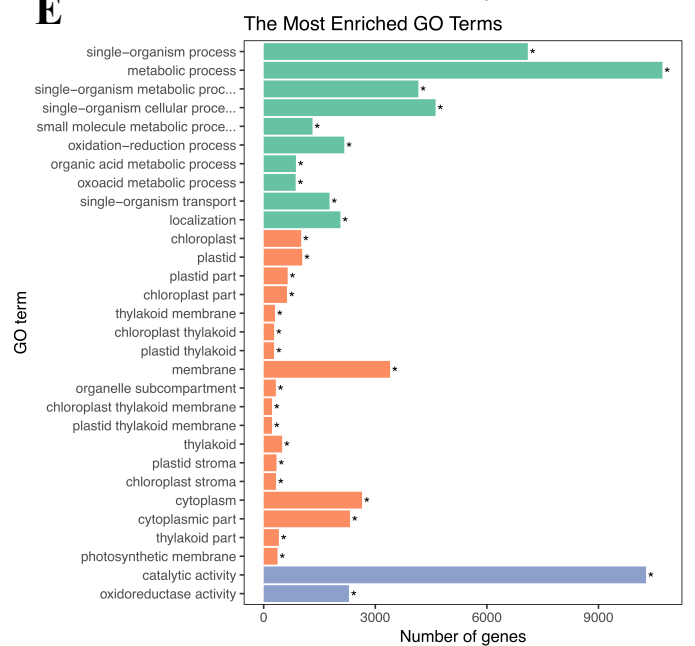

Supplement: Supplementary file 6 — Additional file 6. GO enrichment analysis of all of the DEGs of wheat leaves in response to S. graminum feeding at 2 (A), 6 (B), 12 (C), 24 (D) and 48 hpi (E). [file 12864_2020_6743_MOESM6_ESM.pdf]
